# Similar Events but Contrasting Impact: Appraising the Global Digital Reach of World Heart Day and Atrial Fibrillation Awareness Month

**DOI:** 10.5334/gh.1212

**Published:** 2023-06-13

**Authors:** Kashish Malhotra, Vandita Lal, Monil Majmundar, Ashish Kumar, Gurpreet Singh Wander, Ankur Kalra

**Affiliations:** 1 Department of Medicine, Dayanand Medical College and Hospital, Punjab, India; 2 Department of Cardiology, University of Kansas Medical Center, Kansas City, KS, USA; 3 Department of Internal Medicine, Cleveland Clinic Akron General, Ohio, USA; 4 Department of Cardiology, Dayanand Medical College and Hospital, Punjab, India; 5 Department of Cardiology, Kalra Hospitals, New Delhi, India

**Keywords:** World Heart Day, Atrial Fibrillation Awareness Month, healthcare awareness events, awareness campaign, social media

## Abstract

**Background:**

With over 18.6 million deaths annually, cardiovascular diseases (CVDs) are the leading cause of mortality worldwide. One such complication of CVDs that can result in stroke is atrial fibrillation (Afib). As part of global outreach and awareness, World Heart Day and Atrial Fibrillation Awareness Month are celebrated annually on 29 September and the month of September, respectively. Both of these events are important cardiovascular awareness initiatives to assist public education and develop awareness strategies, and they have received considerable support from leading international organizations.

**Objective:**

We studied the global digital impact of these campaigns via Google Trends and Twitter.

**Methods:**

We evaluated the overall number of tweets, impressions, popularity and top keywords/hashtags, and interest by region to determine the digital impact using various analytical tools. Hashtag network analysis was done using ForceAtlas2 model. Beyond social media, Google Trends web search analysis was carried out for both awareness campaigns to examine ‘interest by region’ over the past five years by analyzing relative search volume.

**Results:**

#WorldHeartDay and #UseHeart (dedicated social media hashtags for World Heart Day by the World Heart Federation) alone amassed over 1.005 billion and 41.89 million impressions as compared with the 1.62 million and 4.42 million impressions of #AfibMonth and #AfibAwarenessMonth, respectively. On Google Trends web search analysis, the impact of Afib awareness month was limited to the USA, but World Heart Day had a comparatively global reach with limited digital involvement in the African continent.

**Conclusions:**

World Heart Day and Afib awareness month present a compelling case study of vast digital impact and the effectiveness of targeted campaigning using specific themes and keywords. Though the efforts of the backing organizations are commended, planning and collaboration are needed to further widen the reach of Afib awareness month.

With over 18.6 million deaths annually, cardiovascular diseases (CVDs) are the leading cause of mortality worldwide [1]. One such complication of CVDs that can result in stroke is atrial fibrillation (Afib), which is expected to affect more than 12.1 million individuals in the US alone by 2030 and increases morbidity and mortality [2]. In order to achieve the UN’s goal of decreasing global mortality from non-communicable diseases (NCDs) by 25% by 2025, it is crucial to raise knowledge about the prevention and management of various CVDs as well as to support research to improve outcomes. As part of global outreach and awareness, World Heart Day and Atrial Fibrillation Awareness Month are celebrated annually on 29 September and the month of September, respectively. Both of these events are important cardiovascular awareness initiatives to assist public education and develop awareness strategies, and they have received considerable support from leading international organizations and the US government, among others. We studied the global digital impact of these campaigns via Google Trends and Twitter without any language or geographical restrictions.

We evaluated the overall number of tweets, impressions, popularity and top keywords/hashtags, and interest by region to determine the digital impact of a healthcare awareness event that has previously been used in comparable research [3,4,5]. Using Sprout Social, we extracted the total number of tweets published in September from 2014 through 2022 for several search queries pertaining to World Heart Day and Afib Awareness Month [6]. Total impressions, which essentially represent the total number of Twitter users a tweet may have reached, were computed using Symplur’s machine learning algorithm [7]. Using SocioViz, real-time analysis of the most popular and recent tweets was done to determine the top connected hashtags and keywords in order to better comprehend the context of the Twitter posts on 30 September [8]. Each hashtag in a hashtag network analysis was represented as a node and connected to other hashtags if they appeared in the same tweet. Different groups of arguments that frequently concur were represented by different colors using ForceAtlas2 model to spatialize the network. Beyond social media, Google Trends web search analysis was carried out for both awareness campaigns to examine ‘interest by region’ over the past five years by analyzing relative search volume.

The results of the study noted that the #WorldHeartDay and #UseHeart (dedicated social media hashtags for World Heart Day by the World Heart Federation) alone amassed over 1.005 billion and 41.89 million impressions compared with the 1.62 million and 4.42 million impressions of #AfibMonth and #AfibAwarenessMonth, respectively. Seven of the top 10 influencers of #WorldHeartDay with the highest impressions were news media outlets followed by WHO and other international organizations. Five of the top 10 influencers of #AfibAwarenessMonth were individual accounts followed by cardiology organizations and pharmaceutical company accounts.

Similarly, over 150,000 tweets (25% year-on-year increase) were posted about World Heart Day compared with the 980 tweets (41% year-on-year decrease) specific to Afib Awareness Month in 2022 as shown in Table 1. On network analysis (Figure 1), the top five keywords associated with Afib Awareness Month were ‘heart,’ ‘rhythm,’ ‘patient,’ ‘amiodarone,’ and ‘stroke.’ The top five associated hashtags with Afib Awareness Month were ‘#cardiotwitter,’ ‘#medtwitter,’ ‘#cardiology,’ ‘#epeeps,’ and ‘#meded.’ On Google Trends web search analysis, the impact of Afib Awareness Month was limited to the USA, but World Heart Day had a comparatively global reach with limited digital involvement in the African continent as shown in Supplementary Figure 1.

**Table 1:** Total number of tweets. For search queries 1 and 2, the time frame was the whole month of September (1–30 September). The time frame for search queries 3 and 4 was 28–30 September. Search query 1- ‘afib OR #afib OR atrial fibrillation OR #atrialfibrillation.’ Search query 2- ‘#afibawarenessmonth OR #afibawareness OR afib awareness OR afib awareness month OR atrial fibrillation awareness OR atrial fibrillation awareness month OR #atrialfibrillationawareness OR #atrialfibrillationawarenessmonth.’ Search query 3- ‘use heart OR #useheart.’ Search query 4- ‘world heart day OR heart day OR #whd OR whd OR #worldheartday OR #heartday.’


	ATRIAL FIBRILLATION TWEETS (SEARCH QUERY 1)	TWEETS SPECIFC TO AFIB AWARENESS MONTH (SEARCH QUERY 2)	TWEETS SPECIFIC TO WORLD HEART DAY DEDICATED KEYWORD – #USEHEART OR USE HEART (SEARCH QUERY 3)	TWEETS SPECIFIC TO WORLD HEART DAY (SEARCH QUERY 4)

2014	7,475	549	3,148	31,996

2015	6,996	613	2,599	56,927

2016	10,332	837	7,466	86,820

2017	11,503	2,348	3,361	61,169

2018	21,906	2,112	5,176	1,20,707

2019	14,150	2,082	6,772	69,790

2020	13,456	1,791	23,578	1,48,823

2021	15,759	1,660	14,900	1,24,668

2022	12,905	980	16,287	1,55,797


**Figure 1:**
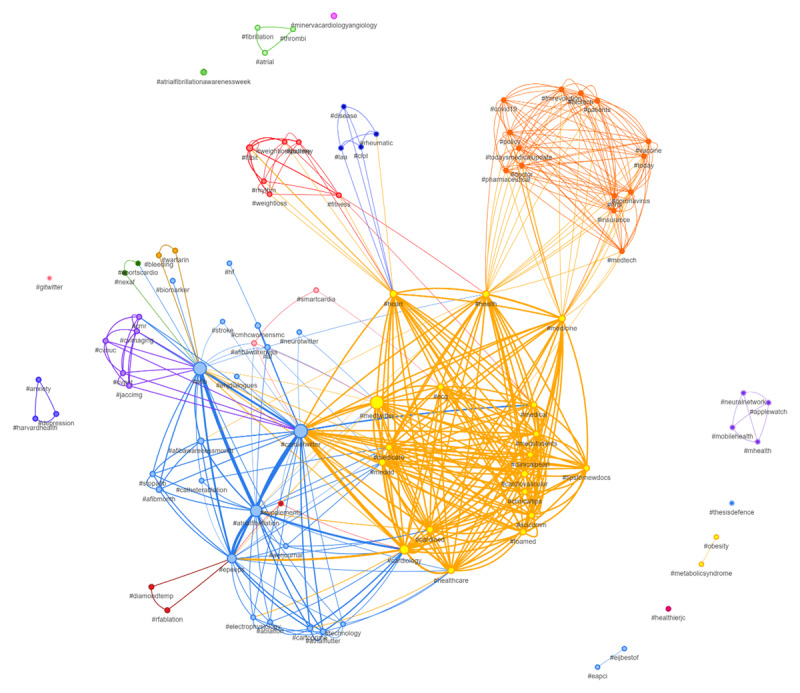
Hashtag network analysis of the search query- ‘afib OR #afib OR atrial fibrillation OR #atrialfibrillation.’

World Heart Day and Afib Awareness Month present a compelling case study of vast digital impact and the effectiveness of targeted campaigning using specific themes and keywords. One may intuitively think that an ‘awareness month’ may have more impact compared with an ‘awareness day,’ simply because it is a month-long event with more cumulative reach than a single-day campaign. However, we reported otherwise in the present study. Though the efforts of the sponsoring organizations are commended, planning and collaboration are needed to further widen the reach of Afib Awareness Month. This directs the conversation to scientifically evaluate the impact of these campaigns and gives all the concerned stakeholders a roadmap to plan ahead. Participation of underrepresented African populations and promoting diversity and inclusion are needed, as has also been noted in earlier work analyzing other awareness months [9,10]. It further reflects that along with getting recognition from governmental bodies or medical societies, social media has the potential to host a successful advocacy campaign with unparalleled reach to form pragmatic communities. Importantly, a blueprint for synergistic cross-platform digital promotion of similar awareness campaigns may be considered. However, in a myriad of awareness campaigns, there seems insufficient evidence to demonstrate the real-life positive impact brought by some of these campaigns. Nonetheless, these events pack the potential to propagate a positive social environment to steer policy development.

## Additional File

The additional file for this article can be found as follows:

10.5334/gh.1212.s1 Supplementary figure 1. Global Google web search interest of World Heart Day and Afib Awareness Month over the past five years.
