# Supplementary material for: Similar Events but Contrasting Impact: Appraising the Global Digital Reach of World Heart Day and Atrial Fibrillation Awareness Month
Source: Glob Heart. 2023 Jun 13;18(1):30. doi: 10.5334/gh.1212 (PMC10275186; doi:10.5334/gh.1212)

Supplementary figure 1. Global Google web search interest of World Heart Day and Afib Awareness Month over the past five years.

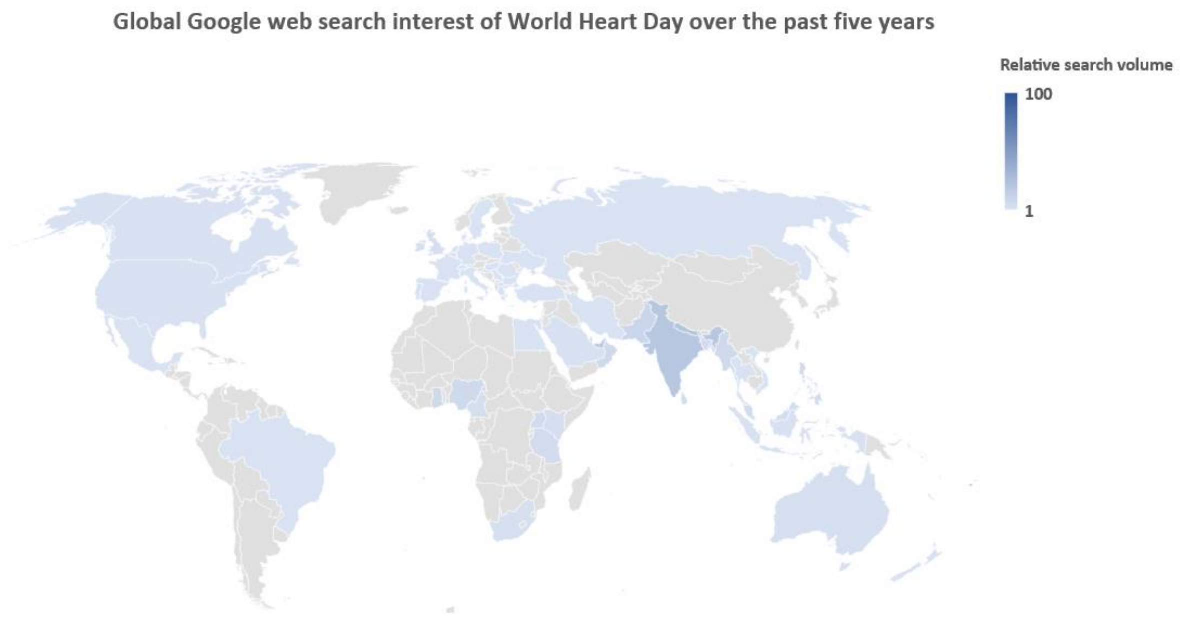

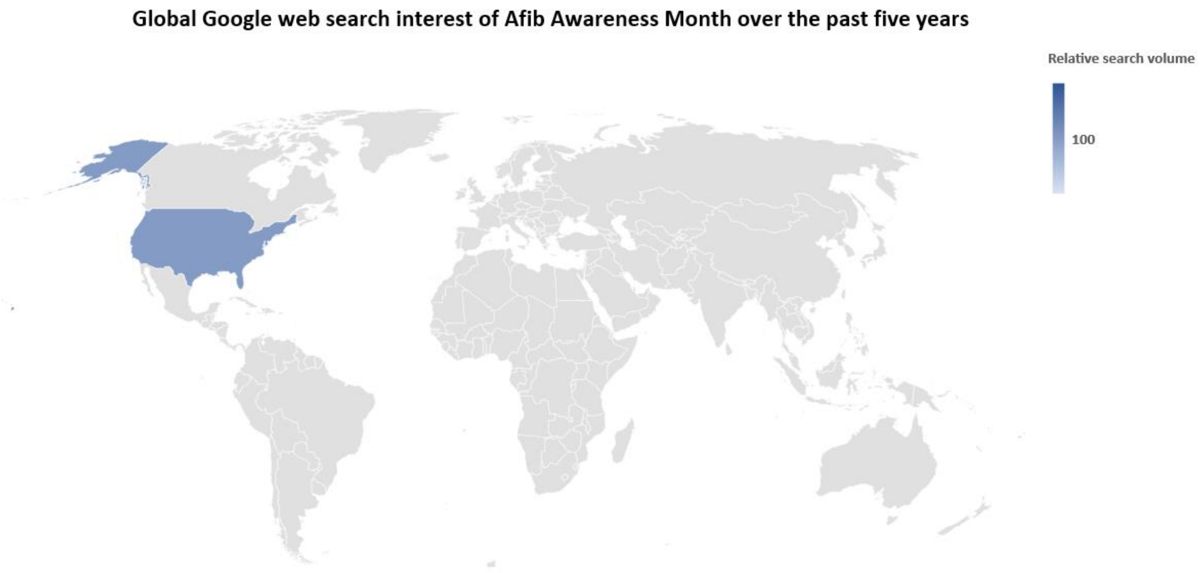

Supplement: Supplementary figure 1. — Global Google web search interest of World Heart Day and Afib Awareness Month over the past five years. [file gh-18-1-1212-s1.pdf]
